# The Combination of *Nigella sativa* Oil‐Based Nanoemulsion and Quercetin Alleviates Oxidative Stress From Exogenous Testosterone Through Antioxidant and Anti‐Inflammatory Mechanisms in Rats

**DOI:** 10.1002/vms3.70984

**Published:** 2026-07-16

**Authors:** Majid Najafi, Negar Panahi, Saeed Hesaraki, Ghasem Akbari

**Affiliations:** ^1^ Department of Veterinary Basic Sciences Science and Research Branch Islamic Azad University Tehran Iran; ^2^ Department of Veterinary Pathobiology Science and Research Branch Islamic Azad University Tehran Iran; ^3^ Department of Veterinary Clinical Sciences Science and Research Branch Islamic Azad University Tehran Iran

**Keywords:** chronic restraint stress, high‐intensity exercise, *Nigella sativa*, quercetin, testicular damage

## Abstract

**Background:**

Exogenous testosterone therapy induces testicular oxidative stress and inflammation, suppressing spermatogenesis. Chronic restraint and high‐intensity exercise stress exacerbate reproductive dysfunction. Quercetin (Qu) and black seed oil (BSO) possess potent antioxidant and anti‐inflammatory properties.

**Objectives:**

This study evaluates the efficacy of Qu‐loaded BSO (Qu‐BSO) nanoemulsion in mitigating testosterone‐induced testicular damage in rats subjected to exercise and restraint stress, as well as to testosterone and finasteride administration.

**Methods:**

A Qu‐BSO nanoemulsion was prepared and characterized. Thirty‐six male Wistar rats were divided into six groups (*n* = 6): control (C), testosterone (T) at 20 mg/kg weekly via subcutaneous injection, testosterone + finasteride (TF) at 1 mg/kg 5 days a week, testosterone + high‐intensity treadmill exercise (TE) for 50 min daily, testosterone + chronic restraint stress (TI) for 3 h daily and testosterone + Qu‐BSO (TQBSNE) for 6 weeks. Serum testosterone, LH, testicular antioxidants (SOD, CAT and GPx), lipid peroxidation (MDA), inflammatory cytokines (TNF‐α and IL‐1β mRNA), and histopathology were assessed.

**Results:**

The T, TF and TI groups exhibited significant testicular damage with elevated oxidative stress and inflammation. The T and TF groups exhibited the highest serum testosterone. TE showed partial protection. The TQBSNE treatment group provided superior protection, with significantly higher SOD, CAT and GPx activities, the lowest MDA levels and reduced TNF‐α and IL‐1β expression. TQBSNE also partially restored LH levels.

**Conclusions:**

The high‐intensity exercise group showed a partial protective effect. Chronic restraint stress exacerbated testicular damage caused by testosterone. Qu‐BSO nanoemulsion effectively counteracted testosterone‐induced testicular damage through antioxidant and anti‐inflammatory mechanisms, representing a promising therapeutic strategy.

## Graphical abstract

Exogenous testosterone and stress can cause severe oxidative stress and inflammation in the testicles. To combat testicular damage caused by testosterone and stress, we developed a nanoemulsion containing quercetin and black seed oil. This innovative formulation significantly enhanced antioxidant defences, reduced inflammation and prevented histological damage, showing improved effectiveness compared to individual treatments.

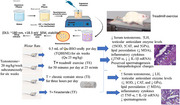

## 1 Introduction

Approximately 30% of infertility cases are due to male factor issues (Ohlander et al. 2016). Testosterone supplementation can increase testicular stress and disrupt the pro‐oxidant/antioxidant balance in adolescent male rats (Pini et al. 2021). It suppresses luteinizing hormone (LH) secretion from the pituitary, thereby reducing endogenous hormone production and intratesticular testosterone levels, which negatively affects spermatogenesis (O'Hara and Smith 2015). As a result, exogenous testosterone may function as a contraceptive, leading to significantly lower sperm counts in men and potentially contributing to male infertility (O'Hara and Smith 2015). Numerous studies have shown that high levels of externally administered testosterone can disrupt sperm production (Ramaswamy and Weinbauer 2014). However, testosterone supplementation for infertility and bodybuilding is quite common (Abdallah et al. 2025). According to the 2019 European Association of Urology (EAU) guidelines on male infertility, testosterone replacement is still strongly discouraged for men who are planning to become parents and for the treatment of male infertility associated with low levels of LH and FSH. Conversely, some studies have shown that high doses of testosterone can negatively influence semen quality due to mechanisms related to oxidative stress and inflammation (O'Hara and Smith 2015; Ohlander et al. 2016; Ramaswamy and Weinbauer 2014). The use of anabolic‐androgenic steroids, with exogenous testosterone, is prevalent among athletes to improve muscle mass and physical performance, as well as among non‐athletes aiming to boost their appearance. Excessive use of testosterone and anabolic steroids has been shown to have adverse effects on the male reproductive system, as indicated by previous studies (El Osta et al. 2016; Rizzuti et al. 2023). Supraphysiological testosterone (10 mg/kg for 30 days) induces the release of proinflammatory cytokines (IL‑1β and IL‐18). It contributes to vascular inflammation, oxidative stress, endothelial dysfunction and alterations in vascular tone and remodelling in C57Bl/6J mice (Alves et al. 2020).

Chronic stress is a key factor in disorders of the hypothalamic‐pituitary‐adrenal axis, resulting in reproductive dysregulation and reduced testosterone levels. Moderate‐intensity exercise may help reverse obesity‐ and age‐related defects in spermatogenesis. On the other hand, some studies have shown that high‐intensity exercise can reduce testosterone levels in both animals and humans (Kelestimur et al. 2021). Exogenous testosterone administration in humans and animals (dogs and horses) modifies testicular histology and physiology (Mason 2023), and these changes are further exacerbated by stress. In this study, we compared the effects of acute stress (treadmill exercise), chronic stress (restraint stress) and oxidative stress induced by testosterone and finasteride consumption on testicular tissue.

BS oil is recognized for its antioxidant, anti‐inflammatory, analgesic and antihistamine properties. It notably improves male reproductive health by enhancing sperm count, motility, morphology and seminal fluid quality (Mahdavi et al. 2015). Key benefits include increased seminal vesicle weight, elevated testosterone levels and reduced sperm morphological abnormalities (Kolahdooz et al. 2014). Thymoquinone, a compound found in BS oil, protects against oxidative damage caused by various chemicals, thereby safeguarding sperm and seminal fluid, and increasing the number of Leydig cells in the testes (Saeed et al. 2013).

Qu is a noteworthy flavonoid with significant potential for various health applications due to its diverse biological activities, including antioxidant (Oyovwi et al. 2025), anti‐inflammatory (Oghenetega et al. 2024) and anticancer effects. However, its poor bioavailability challenges its effectiveness as a nutraceutical (Okselni et al. 2024).

Nanoemulsifying drug delivery systems enhance drug absorption by increasing membrane fluidity, bypassing the first‐pass effect and improving oral solubility and bioavailability. Studies indicate that Qu‐nanoemulsion is particularly effective for drug delivery owing to its micronization, thermal stability, storage stability and enhanced bioavailability (Alharbi et al. 2025; Mahadev et al. 2022; Okselni et al. 2025).

Based on the potent individual properties of Qu and BS oil, we hypothesized that a novel nanoemulsion combining both compounds (Qu‐BSO) would synergistically protect against testicular damage induced by exogenous testosterone and stress.

We expect that the Qu‐BSO nanoemulsion will significantly mitigate hormonal imbalance, oxidative stress, inflammatory cytokine expression and histopathological damage, ultimately preserving testicular function. This study addresses a critical gap by proposing a novel nano‐formulation as a promising therapeutic strategy against iatrogenic and stress‐related testicular impairment.

## 2 Materials and Methods

### 2.1 Materials

Crude BS oil was obtained from the Research Institute of Medicinal Plants and Raw Materials, Faculty of Pharmacy, Shahid Beheshti University. The supplier analytically standardized the oil to contain a minimum of 0.8% (w/w) thymoquinone, as determined by high‐performance liquid chromatography (HPLC), to ensure batch‐to‐batch consistency and biological activity. Qu, polyethylene glycol (PEG) 400, and Tween 80 were obtained from Sigma Aldrich (St. Louis, MO, USA). Testosterone enanthate (100 mg/mL) ampoules were supplied by Chemical Industries Abu Rayhan (Iran). Sesame oil was used as a vehicle to dilute the testosterone enanthate.

### 2.2 Qu‐BSO Formulation

Based on our previous research, a homogenous mixture was formed with some modifications (Jafari et al. 2024). The process involved mixing the following components: 1% Qu, 10% BS oil, 30% Tween 80 (a surfactant that enhances the solubility of Qu and stabilizes the nanoemulsion) and 10% PEG 400 (a cosurfactant that improves emulsification efficiency and reduces droplet size). Tween 80 is a commonly used nonionic surfactant considered safe for oral administration in pharmaceutical formulations. This mixture was stirred for 10 min at 50°C. The aqueous phase (deionized water, preheated to 50°C) was added dropwise to the oil phase under continuous stirring until the final volume was 100 mL. After cooling to room temperature, the coarse emulsion was subjected to high‐energy emulsification using a probe sonicator (20 kHz, 1500 W, Sonics, Newtown, CT, USA) for 10 min (Zhang et al. 2020). Following this process, an oil‐in‐water (O/W) nanoemulsion drug delivery system was formed and stored at 4°C.

### 2.3 The Stability Tests

Qu‐BSO aqueous dispersions were evaluated for their ability to emulsify and clarify spontaneously. The consistency of the formulations was assessed through three steps. In the first step, the formulation was diluted with an aqueous medium. The mixture was centrifuged at 15000 rpm for 15 min, and no phase separation was observed. The second and third steps involved freeze‐thaw and heating‐cooling cycles. For the freeze‐thaw cycle, the mixture was diluted with deionized water at a 1:50 ratio and stored at −20°C for 2 days. Subsequently, it was thawed, and any phase separation was investigated. The mixture was maintained at 40°C for 2 days during the heating‐cooling cycle, then cooled and reassessed for phase separation. No phase separation was observed. Quantitatively, particle size and polydispersity index (PDI) remained stable over 4 weeks at both 4°C, 25°C and 40°C, with a mean size variation of less than 10 nm and a PDI change of < 0.1.

### 2.4 Vesicular Size and Zeta Potential

The vesicular size, zeta potential and mean diameter of the BS oil nanoemulsion were evaluated using dynamic light scattering (DLS) with a Nano‐ZS SZ‐100 (Horiba, Japan). Samples were diluted 1:100 in deionized water and measured in triplicate at 25°C. Results are presented as mean diameter ± standard deviation (SD).

### 2.5 QU‐BSO Examination by Scanning Electron Microscopy

QU‐BSO morphology was examined using scanning electron microscopy (SEM) with a TESCAN Vega 3 (TESCAN SEM, Czech Republic). Samples were prepared by drop‐casting onto aluminium stubs, air‐drying and sputter‐coating with a 10 nm gold layer. Imaging was performed at an accelerating voltage of 15 kV.

### 2.6 Experimental Study

Ten‐week‐old male Wistar rats, weighing between 180 and 200 g, were housed in an air‐conditioned room at a temperature of 22 ± 2°C and 55 ± 5% humidity. The rats were kept on a 12‐h light/dark cycle. This study was approved by the National Committee on Ethics in Biomedical Research (ethics.research.ac.ir) of the Islamic Azad University, Science and Research Branch (approval ID IR.IAU.SRB.REC.1401.339). Moreover, in accordance with the Helsinki Declaration, this research was conducted with due care to protect the welfare of the animals involved. The rats were given a 1‐week acclimatization period before the experiments began. The 36 rats were equally divided into six groups: control group (C), testosterone enanthate group (T), testosterone enanthate plus finasteride group (TF), testosterone enanthate plus exercise group (TE), testosterone enanthate plus restraint stress group (TI), testosterone enanthate plus Qu in BS oil group (TQBSNE). All groups, except the control group, received subcutaneous injections of testosterone enanthate at a dosage of 20 mg/kg weekly for 6 weeks (Joksimović et al. 2017). The C group received subcutaneous injections of the testosterone vehicle (sesame oil) and oral administration of the nanoemulsion vehicle (deionized water), following the same schedule as the treatment groups. The TF group received 1 mg/kg finasteride orally for 5 days a week. The exercise protocol consisted of treadmill exercise performed 5 days a week for 6 weeks (50 min at 25 m/min), for the TE group (Kelestimur et al. 2021). The restraint protocol was performed using transparent plastic rat restrainers for 3 h, 5 days per week, over 6 weeks, in the TI group (Kelestimur et al. 2021). The treatment group (TQBSNE) received BS oil nanoemulsion (0.5 mL) containing Qu (1%) orally daily (Jafari et al. 2024). This dose delivered approximately 25 mg/kg body weight of Qu (based on a 1% w/v Qu concentration in the formulation and an average rat weight of 200 g).

At 72 h after receiving the final doses, the rats were euthanized using ketamine and xylazine. Serum samples were stored at −20°C for biochemical analysis. The testicular tissue was isolated and divided into two sections: one section was prepared for haematoxylin and eosin (H&E) staining, while the other was stored at −70°C for RNA extraction.

### 2.7 Determination of Superoxide Dismutase, Catalase and Glutathione Peroxidase Enzyme Activity

Activities of superoxide dismutase (SOD), catalase (CAT) and glutathione peroxidase (GPx) enzymes were measured in testicular tissues using a colourimetric kit (SOD: ZX‐44108‐96; CAT: ZX‐44102‐96; GPx: ENZ‐237, ZellX assay kit), following the manufacturer's protocol.

### 2.8 Oxidative Stress Marker

Lipid peroxidation was assessed by measuring malondialdehyde (MDA) levels in testicular tissue using a colourimetric TBARS Assay Kit (ZX‐44116 ‐ ZELLX) according to the manufacturer's instructions.

### 2.9 Serum Levels of Testosterone and LH Hormone

Serum levels of testosterone and LH hormones were measured using commercial ELISA kits (Testosterone: Rat Testosterone [TES] ELISA Kit, catalogue number ZB‐1101‐RT; LH: Rat LH ELISA Kit, catalogue number ZB‐1103‐RT) according to the manufacturer's instructions.

### 2.10 Gene Expression of IL‐1β and TNF‐α

A Qiagen RNeasy kit was utilized to extract total RNA from tissue samples. A reverse transcription kit was used to synthesize complementary DNA (cDNA). The SYBR Green Master Mix kit was used to run qRT‐PCR with the following forward and reverse primers (Table 1):

**TABLE 1:** Gene Expression of IL‐1β and TNF‐α.

	Forward primer	Reverse primer
IL‐1β	5'‐GCA ACT GTT CCT GAA CTC AAC‐3'	5'‐ATC TTT TGG GGT CCG TCA ACT‐3'
TNF‐α	5'‐CCC AGA CCC TCA CAC TCA GAT‐3'	5'‐TGT TGT CTC TTG ATG GCA GAC‐3'
β‐actin	5'‐CCC ATC TAT GAG GGT TAC GCT‐3'	5'‐TTT AAT GTC ACG CAC GAT TTC‐3'

The 2^−ΔΔCt^ method was employed as an internal control, using a housekeeping gene (β‐actin), to assess the expression of IL‐1β and TNF‐α. Fold changes in gene expression between the experimental and control groups were calculated.

### 2.11 Histological Examination

After fixation with formalin, the tissue was sectioned into paraffin‐embedded blocks, stained with H&E and examined using light microscopy. The Johnsen scoring system was employed to histologically classify the seminiferous tubular cross‐section in order to identify spermatogenic cells. A score of 10 on the Johnsen scoring system indicates complete spermatogenesis, characterized by numerous spermatozoa and a germinal epithelium with an organized, regular thickness, resulting in a clear lumen. A score of ‘9’ indicates a significant presence of spermatozoa, although the germinal epithelium shows disorganization with considerable sloughing or obstruction of the lumen. A score of ‘8’ denotes that only a small number of spermatozoa are detected in the sample. A score of ‘7’ indicates the absence of spermatozoa, while many spermatids are present. A score of ‘6’ indicates the absence of spermatozoa, with only a few spermatids present. Finally, a score of ‘5’ indicates the absence of both spermatozoa and spermatids, with many spermatocytes present (Teixeira et al. 2019).

### 2.12 Statistical Analyses

The data were analysed statistically using SPSS version 25. One‐way ANOVA was used to determine differences between groups, followed by post hoc Tukey's test to determine the differences among the groups in hormone levels and antioxidant defence. Analysis results are presented as mean ± standard error of mean (SEM) or SD. Fold changes in gene expression (2^−ΔΔCt^ values) were log‐transformed and analysed using one‐way ANOVA followed by Tukey's post hoc test.

## 3 Results

### 3.1 Overall Graphical Abstract

**FIGURE 1:**
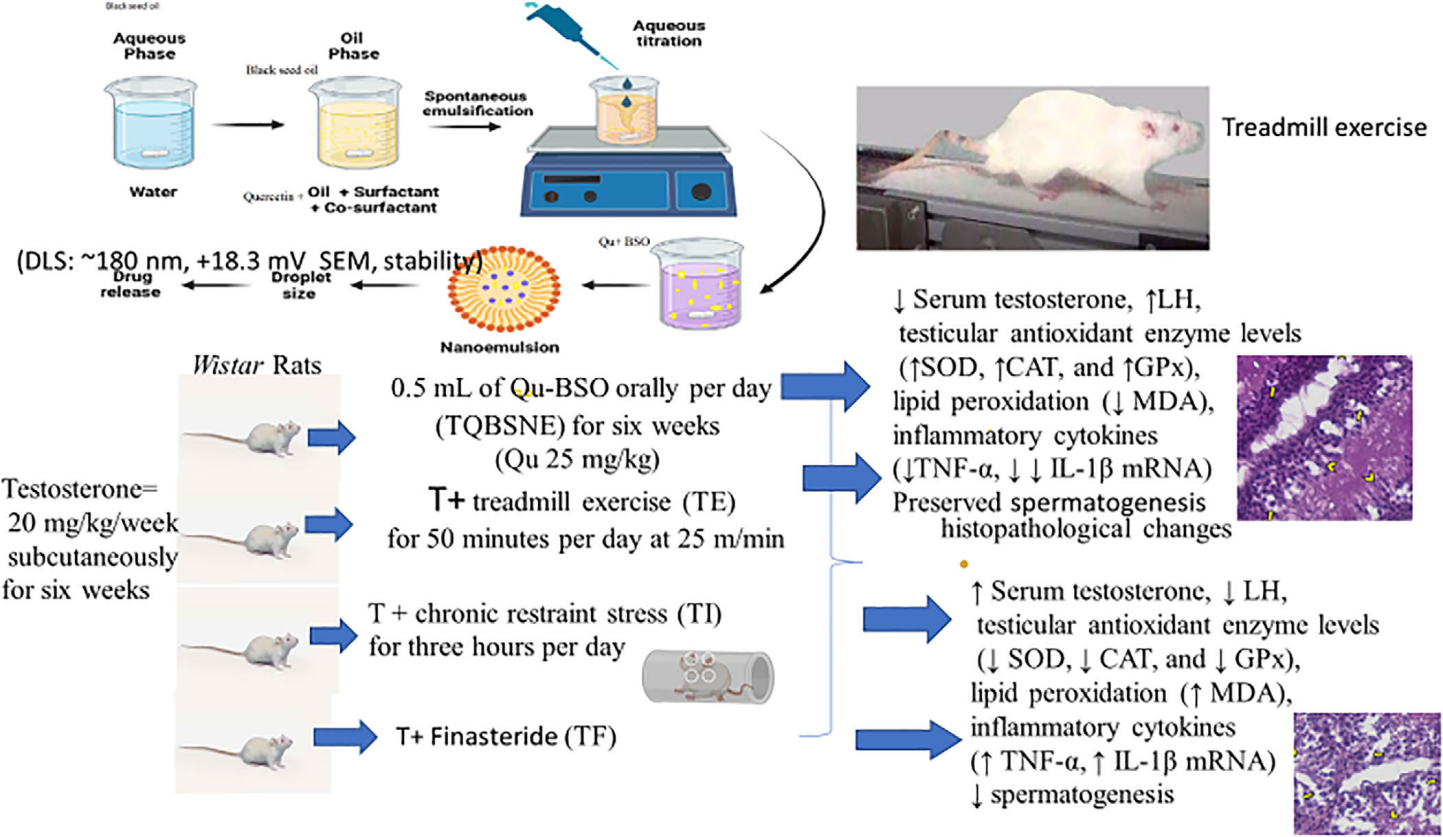
Overall graphical abstract images.

Exogenous testosterone, combined with restraint or exercise stress, induces testicular oxidative stress and inflammation. The novel Qu‐BSO counteracts this damage by enhancing antioxidant defences, suppressing inflammatory cytokines and preserving testicular structure in rats, demonstrating a promising protective strategy (Figure 1).

### 3.2 Vesicular Size and Zeta Potential

**FIGURE 2:**
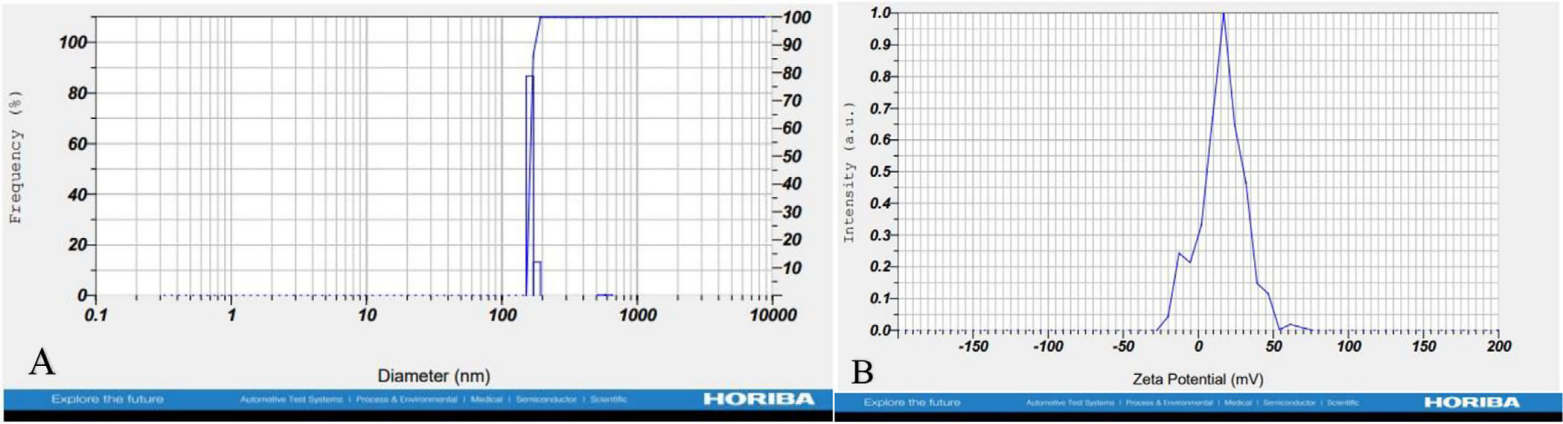
Particle sizes (A) and zeta potential value (B) of *Nigella sativa* seed oil nanoemulsion with quercetin.

The particle sizes were around 180 nm. Furthermore, 90% of the particles fall below 180 nm, indicating a homogenous distribution (Figure 2A). The nanoemulsion particle size is primarily 180 nm, with most particles within this range. The small particle size enhances the emulsion's stability and bioavailability, making it suitable for use in drug delivery applications.

The graph shows a peak at approximately 18.3 mV (Figure 2B). This positive charge can help prevent aggregation by creating repulsive forces between similarly charged particles.

### 3.3 The SEM Image of the Quercetin‐Loaded Black Seed Oil Nanoemulsion

**FIGURE 3:**
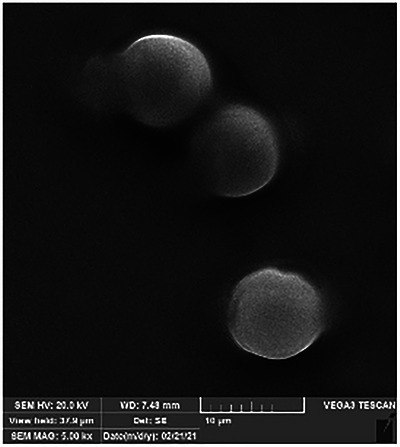
The SEM image of the quercetin‐loaded black seed oil nanoemulsion.

The SEM image depicts spherical particles, a characteristic feature of emulsions. The particles fell within the expected size range for nanoemulsions, typically below 200 nm. The morphology appears relatively uniform, suggesting a consistent formulation process. While SEM images primarily show surface contours, the smooth, spherical appearance of the particles can indicate a well‐formed emulsion with minimal aggregation or irregularities (Figure 3).

### 3.4 Hormone Levels

**FIGURE 4:**
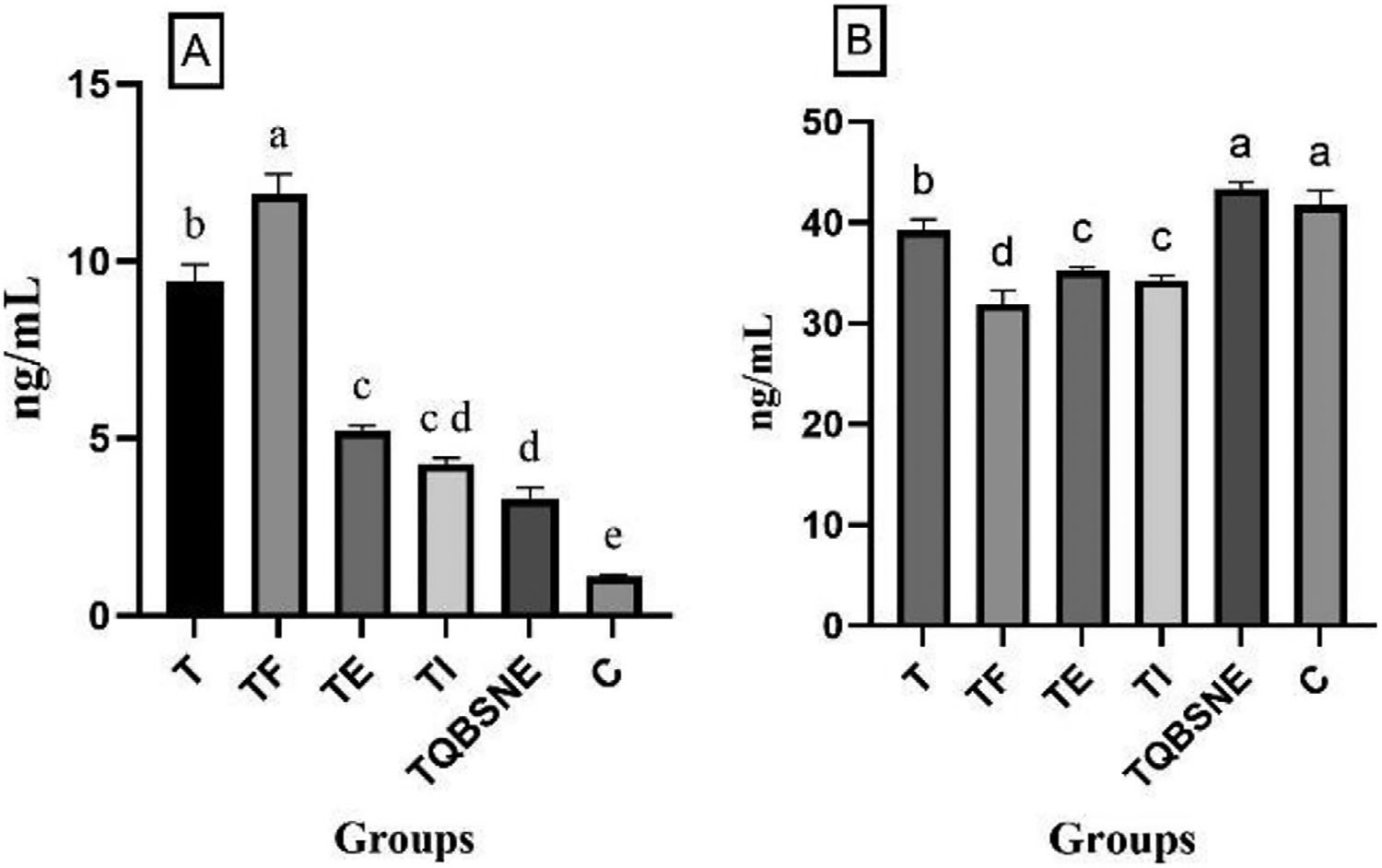
Serum testosterone levels (A), serum LH level (B), C: control, T: testosterone, TF: testosterone + finasteride, TE: testosterone + exercise, TI: testosterone + restraint, TQBSNE: testosterone + quercetin + black seed oil‐based nanoemulsion. Data were analysed by one‐way ANOVA followed by Tukey's test. Values are mean ± SEM (*n* = 6). Different superscript letters (a, b, c, d and e) indicate significant differences (*p* < 0.05) between groups.

The TF group exhibited the highest serum testosterone level (approximately 12 ng/mL) and the lowest LH level (31.9 ± 0.5), probably due to the administration of testosterone and finasteride (Figure 4). The T group's testosterone level was slightly lower than that of the TF group. Finasteride inhibits 5α‐reductase, thereby blocking the conversion of testosterone to DHT. This reduces testosterone clearance, leading to its accumulation in the serum. In the TI, TE and TQBSNE groups, testosterone levels were lower than in the T and TF groups. The TE group showed decreased testosterone levels, suggesting that treadmill exercise may exert protective effects, possibly by enhancing antioxidant defence mechanisms. The TQBSNE group exhibited reduced testosterone levels; however, Qu and BS oil might have had an additive or synergistic effect on oxidative stress. The lowest LH levels were observed in the TF group compared to all groups (*p* < 0.05), while the highest LH levels were observed in the control and TQBSNE groups (*p* < 0.05).

### 3.5 Testicular Antioxidant Enzymes (SOD, CAT and GPx) and Lipid Peroxidation (MDA)

**FIGURE 5:**
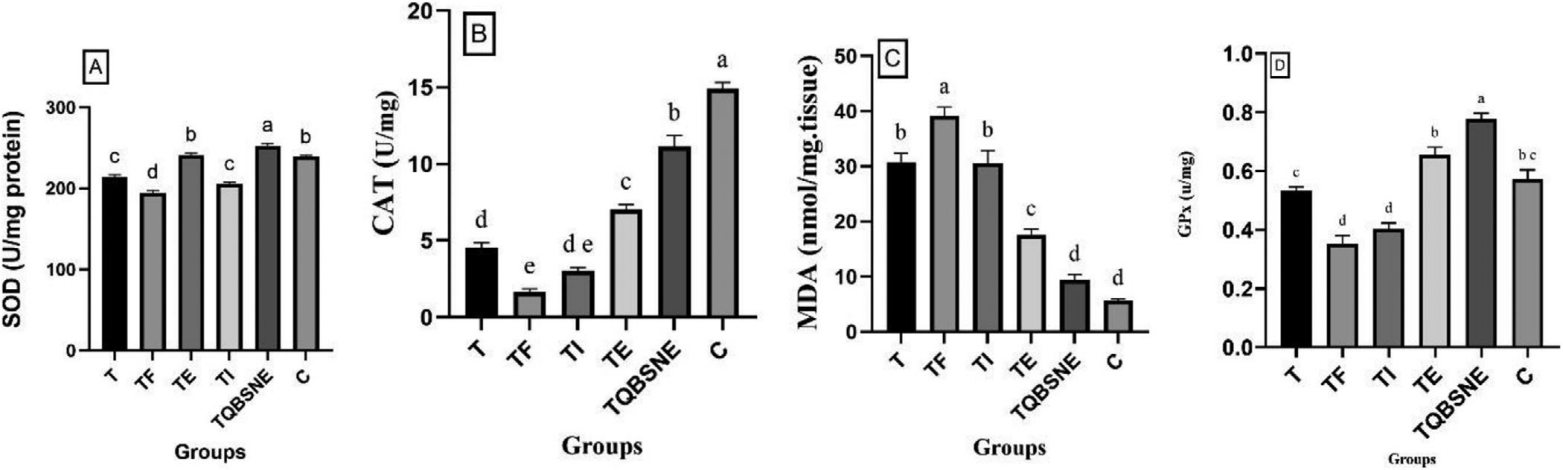
The superoxide dismutase (SOD) activity (A), catalase (CAT) activity (B), the malondialdehyde (MDA) level (C), the glutathione peroxidase (GPx) activity (D), C: control, T: testosterone, TF: testosterone + finasteride, TE: testosterone + exercise, TI: testosterone + restraint, TQBSNE: testosterone + quercetin + black seed oil‐based nanoemulsion. Data were analysed by one‐way ANOVA followed by Tukey's test. Values are mean ± SEM (*n* = 6). Different superscript letters (a, b, c, d and e) indicate significant differences (*p* < 0.05) between groups.

CAT, SOD and GPx activities in the TQBSNE group were enhanced, indicating the most effective combined treatment with Qu and a BS oil‐based nanoemulsion (Figure 5A,B,D) compared to the T, TI and TF groups (*p* < 0.05). The lowest activity was observed in the T, TI and TF groups (*p* < 0.05), indicating significant oxidative stress. The T, TI and TF groups showed elevated lipid peroxidation. The TE group was lower than the T, TI and TF groups, suggesting that treadmill exercise protects against oxidative damage. The TQBSNE group had the lowest lipid peroxidation levels, indicating the most effective combined treatment (Figure 5C).

### 3.6 The mRNA Expression Levels of TNF‐α and IL‐1β

**FIGURE 6:**
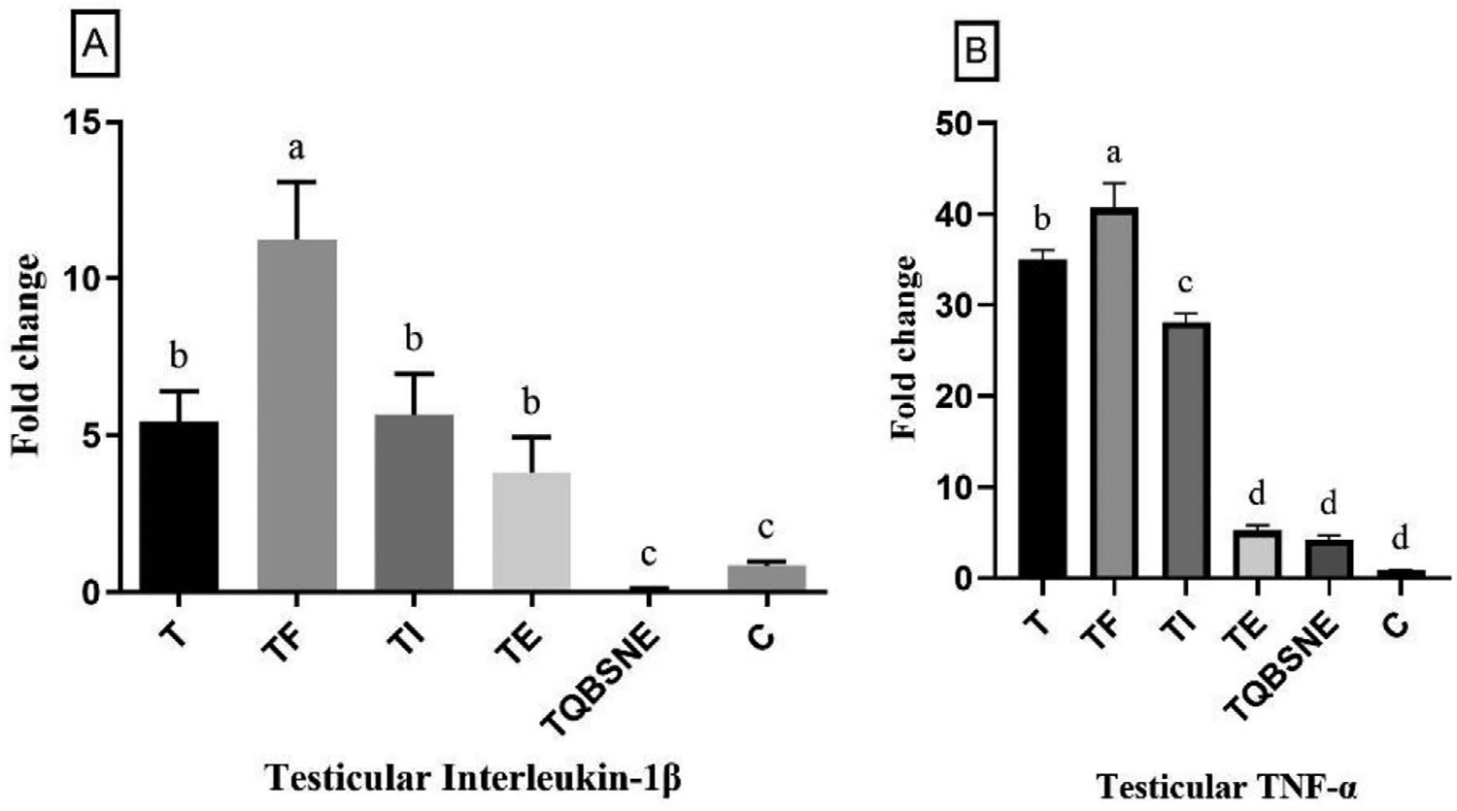
The testicular expression of IL‐1β (A) and TNF‐α (B); values are expressed as the mean ± SD (*n* = 6). C: control, T: testosterone, TF: testosterone + finasteride, TE: testosterone + exercise, TI: testosterone + restraint, TQBSNE: testosterone + quercetin + black seed oil‐based nanoemulsion. Data were analysed by one‐way ANOVA followed by Tukey's test. Different superscript letters (a, b, c and d) indicate significant differences (*p* < 0.05) between groups.

The T and TI groups showed increased IL‐1β (Figure 6A) and TNF‐α (Figure 6B) expression compared to the C group. The TF group showed the highest IL‐1β (11.25 ± 1.83) and TNF‐α (40.65 ± 6.769) expression levels, suggesting that finasteride may have exacerbated testosterone‐induced inflammation (*p* < 0.05). The TQBSNE group demonstrates a significant anti‐inflammatory effect by reducing TNF‐α and IL‐1β expression levels compared with the T, TE and TF groups, providing improved protection against testosterone‐induced inflammation (*p* < 0.05). Exercise (TE group) substantially decreased TNF‐α levels (5.2 ± 1.4), highlighting its anti‐inflammatory properties (*p* < 0.05).

### 3.7 Histopathological Findings

**FIGURE 7:**
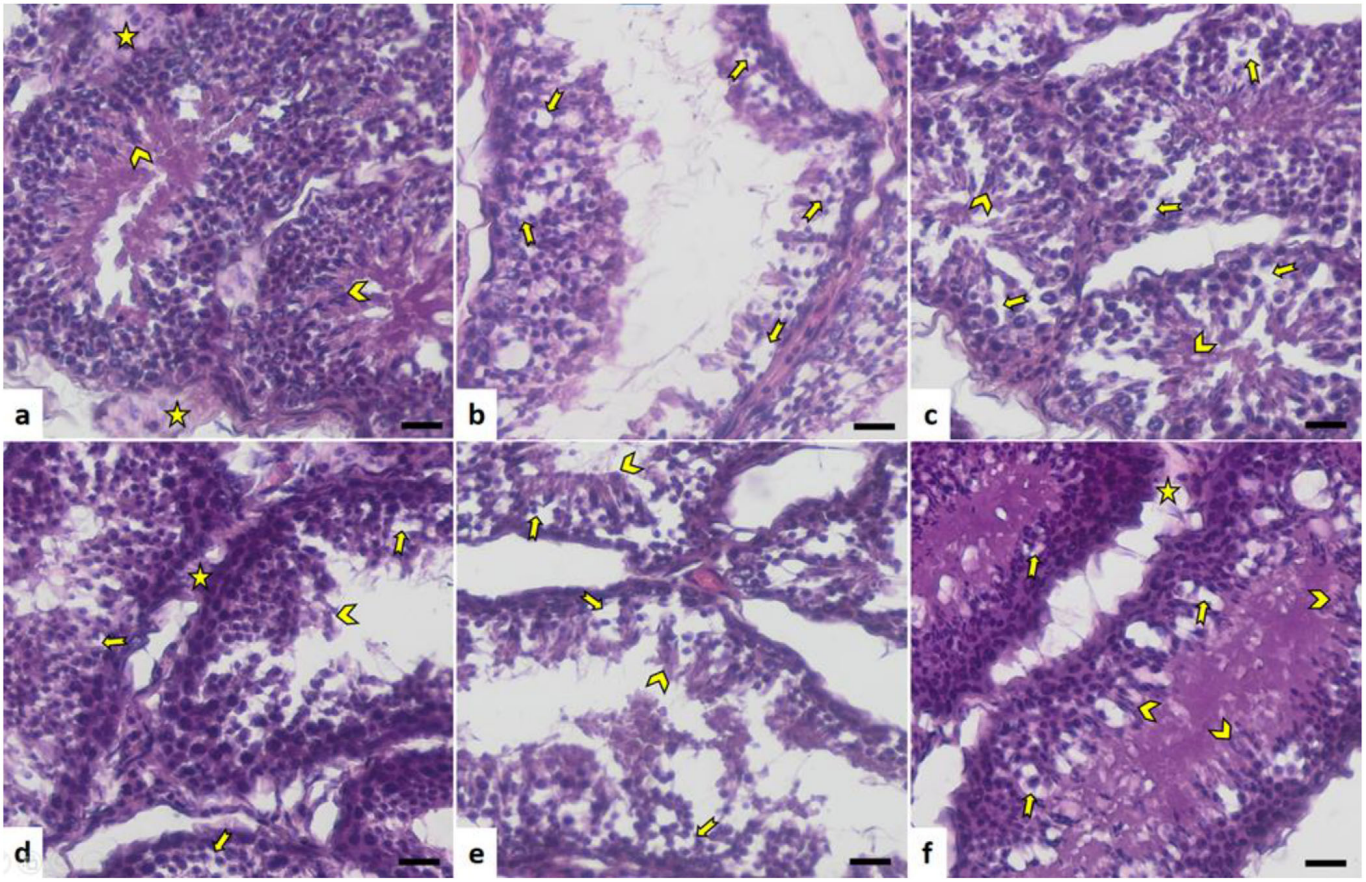
Testes in different experimental groups: the testicular tissue of the control group (a), T: testosterone group (b), TF: testosterone + finasteride, (c), TI: testosterone + restraint (d), TE: testosterone + exercise (e), TQBSNE: testosterone + quercetin + black seed oil‐based nanoemulsion (f). a thick arrow indicates vacuolation of Sertoli cells, a star represents Leydig cells, and the upper arrow highlights spermatogenesis.

**FIGURE 8:**
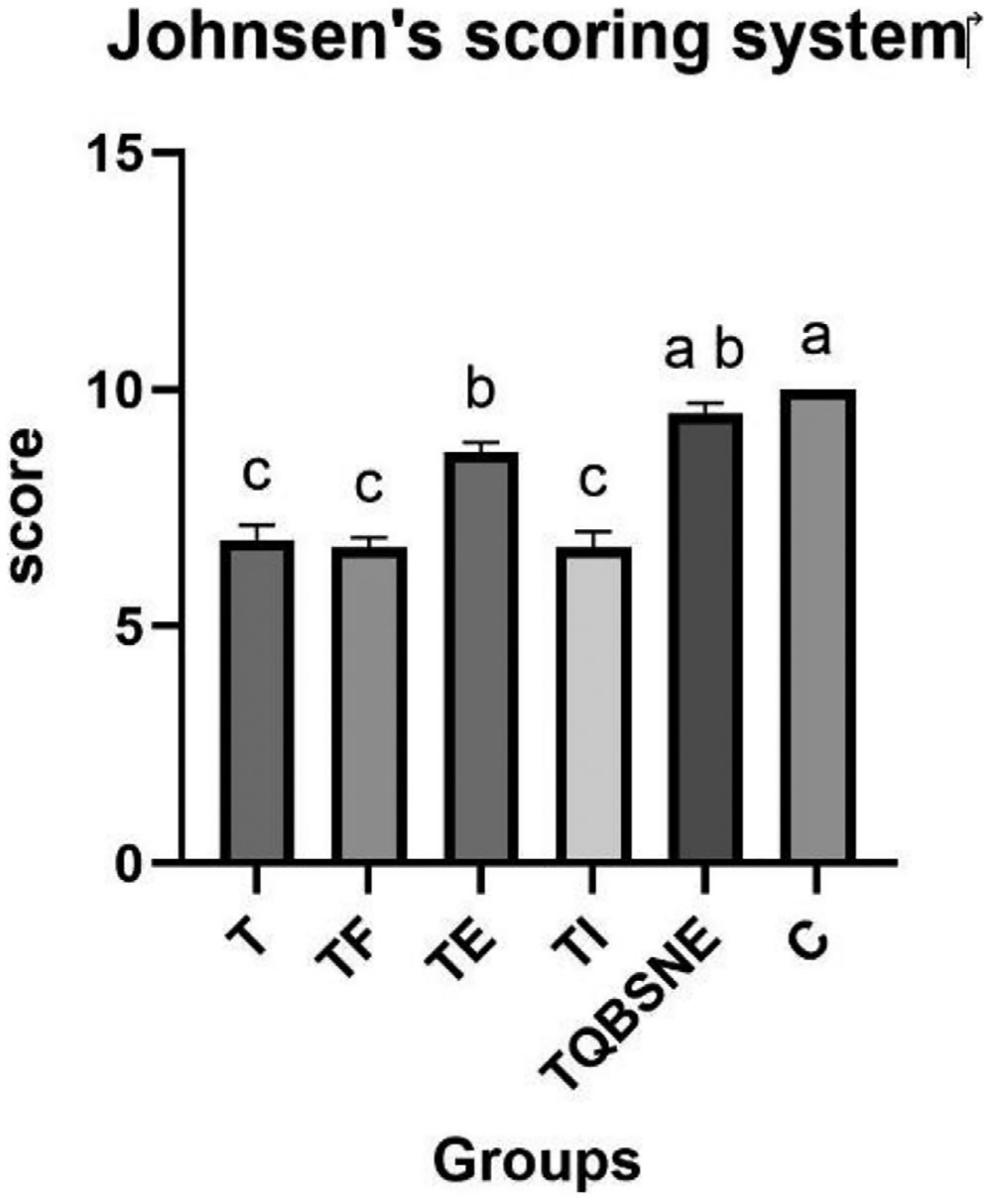
The Johnsen scoring system for evaluating spermatogenesis cells: C: control, T: testosterone, TF: testosterone + finasteride, TE: testosterone + exercise, TI: testosterone + restraint, TQBSNE: testosterone + quercetin + black seed oil‐based nanoemulsion. Data were analysed by one‐way ANOVA followed by Tukey's test. Values are mean ± SEM (*n* = 6). Different superscript letters (a, b and c) indicate significant differences (*p* < 0.05) between groups.

The control group had normal spermatogenic tissue in the testes and a healthy epididymis; the seminiferous tubules were connected and regular, and the Leydig cells were normal. Spermatogonia, spermatocytes and spermatids were also normal (Figure 7a). In the T, TI and TF groups, decreased spermatogenesis, reduced Leydig cell numbers, intertubular oedema and vacuolation of the Sertoli cell cytoplasm were observed, without a marked leukocyte influx (Figure 7b–d). In contrast, in the exercise group, the decrease in Leydig cell number was similar to that in the TQBSNE group (Figure 7f); all tissue parameters showed significant improvement compared with the testosterone group. The Johnsen scoring system was utilized as a semi‐quantitative approach to assess spermatogenic cells (Figure 8). The TQBSNE group exhibited the highest level of complete spermatogenesis, characterized by an organized germinal epithelium and regular thickness (9.500 ± 0.5), in comparison to the T group (6.83±0.7, *p* < 0.0001). The T, TF and TI groups exhibited reduced spermatogenesis compared with the C group (*p* < 0.0001). Exercise improved spermatogenesis (8.7 ± 0.5), with a significant difference in the TE group compared with chronic restraint stress and testosterone‐induced oxidative stress (*p* < 0.0001).

## 4 Discussion

The central finding of this study is that exogenous testosterone administration, particularly in conjunction with finasteride or chronic stress, induces profound testicular damage through interconnected mechanisms of oxidative stress and inflammation. Our results in the T group confirm that supraphysiological testosterone levels provide negative feedback suppression of the HPG axis, leading to reduced LH and, consequently, a critical drop in testicular testosterone necessary for spermatogenesis (Khodamoradi et al. 2021). Beyond this negative feedback mechanism of exogenous testosterone on LH secretion, we demonstrate that the damage is mechanistically driven by a significant increase in oxidative stress, as evidenced by suppression of key antioxidant enzymes (SOD, CAT and GPx) and elevated lipid peroxidation (MDA). This pro‐oxidant environment was associated with a strong inflammatory response, characterized by a significant increase in TNF‐α and IL‐1β levels (Rastrelli et al. 2019). In the TF group, which exhibited the highest cytokine levels, we observed a 40‐fold increase in TNF‐α and a 5‐fold increase in IL‐1β following exogenous testosterone administration. Finasteride, by inhibiting 5α‐reductase and elevating serum testosterone while keeping DHT critically low, appears to induce a distinct and pronounced inflammatory state, as evidenced by the highest observed levels of TNF‐α and IL‐1β. This suggests that DHT may play a crucial function in maintaining testicular immune homeostasis. This is consistent with studies demonstrating that inflammatory cytokines can directly prevent steroidogenic enzymes in Leydig cells, leading to germ cell death (Haffez et al. 2024). Histological examination revealed decreased spermatogenesis, reduced Leydig cell numbers, Sertoli cell vacuolation and interstitial oedema in T, TI and TF groups. Intratesticular testosterone was not directly measured, which is a limitation of the study.

The addition of chronic restraint stress (TI group) exacerbated this damage, likely through activation of the stress axis and the TLR‐4/NF‐κB pathway, further amplifying pro‐inflammatory cytokine production (Kelestimur et al. 2021). In contrast, the high‐intensity exercise group (TE) showed a partial protective effect. While also a stressor, exercise appears to have induced adaptive antioxidant mechanisms, as evidenced by moderate preservation of antioxidant enzyme activities and a significant reduction in inflammatory cytokines compared with the T and TI groups.

In plain contrast to the damaged groups, co‐treatment with the Qu‐loaded BS oil nanoemulsion (TQBSNE) provided comprehensive protection across all measured parameters. The TQBSNE group exhibited the most potent restoration of the antioxidant defence system, demonstrating the highest activities of SOD, CAT and GPx. This indicates a powerful synergistic effect between Qu and BS oil in neutralizing superoxide anions and hydrogen peroxide, thereby mitigating oxidative damage. This is further supported by the lowest MDA level in the TQBSNE group, confirming the adequate protection of cellular membranes from lipid peroxidation (Ahmad et al. 2021; Oyovwi et al. 2025; Oyovwi et al. 2023).

The anti‐inflammatory effects of TQBSNE were equally profound. The formulation dramatically suppressed the testosterone‐ and stress‐induced overexpression of TNF‐α and IL‐1β, thereby reducing their levels to near‐normal levels. This potent effect can be attributed to the combined action of Qu and thymoquinone (the active component of BS oil), both of which are known inhibitors of the NF‐κB signalling pathway (Oghenetega et al. 2024), a master regulator of inflammation (Okselni et al. 2025). The biochemical recovery was directly reflected in the histopathological findings, with the TQBSNE group showing near‐normal testicular architecture, preserved spermatogenesis with an organized germinal epithelium and regular thickness, minimal vacuolation and healthy Leydig cells.

The superior efficacy of the TQBSNE treatment is attributable not solely to the pharmacology of its active compounds but also to the nanoemulsion delivery system. Qu, despite its potent bioactivity, exhibits poor aqueous solubility and low oral bioavailability (Mahadev et al. 2022). The developed O/W nanoemulsion, with its small particle size (∼180 nm) and positive zeta potential (+18.3 mV), directly addresses these limitations. The lipid‐based core enhances the solubility of lipophilic Qu, while the nanoscale droplets increase the surface area for absorption and may facilitate targeted delivery to inflamed tissues (Sah et al. 2023). The physical stability test of the BS oil‐based nanoemulsion demonstrated that it remained stable at room temperature (25 ± 2°C) and at low temperatures (4 ± 2°C) for 8 weeks, indicating that the surfactant concentrations were suitable. The formula included black cumin seed essential oil, known for its antibacterial, antioxidant and antifungal properties. However, at higher temperatures (40 ± 2°C), the nanoemulsion became unstable. Overall, the black cumin seed oil nanoemulsion shows promise for drug delivery, with no significant changes in particle size, consistency, or adsorption efficiency observed over 180 days (Usmani et al. 2019). This formulation strategy ensures that higher concentrations of both Qu and thymoquinone reach the target site, thereby amplifying their synergistic antioxidant and anti‐inflammatory effects (Alsabeelah and Kumar 2023). Combining Qu and BS oil nanoemulsion synergistically enhanced SOD and GPx activity, reducing oxidative stress and improving histological architecture in prostatic tissue, thereby attenuating testosterone‐induced benign prostatic hyperplasia in a rat model (Jafari et al. 2024). This study demonstrates that combining Qu with BS oil nanoemulsion enhances antioxidant defence and reduces testosterone‐induced oxidative stress. Additionally, by forming a stable nanoemulsion, the gastrointestinal absorption of hydrophobic compounds like Qu—known for their low bioavailability—is improved due to an increase in their anti‐inflammatory effects (Alsabeelah and Kumar 2023; Jafari et al. 2024; Sah et al. 2023; Usmani et al. 2019; Zhang et al. 2020). Furthermore, the findings suggest that the side effects associated with testosterone administration can be alleviated through exercise and antioxidant supplementation. It is recommended that future research investigate the simultaneous use of antioxidant compounds alongside high‐intensity exercise to more effectively manage oxidative stress and inflammation.

## 5 Conclusion

In conclusion, these findings demonstrate that a nanoemulsion of Qu and BS oil may have clinical relevance for men receiving testosterone therapy, anabolic steroids, or exposed to chronic stress, particularly in the context of fertility preservation. It outperformed all other interventions, including high‐intensity exercise, by simultaneously reducing oxidative stress and inflammation and preserving spermatogenesis and testicular morphology. The addition of chronic restraint stress exacerbated testosterone‐induced testicular damage and decreased spermatogenesis. The effectiveness of this approach emphasizes the importance of advanced delivery systems in improving the efficacy of lipophilic compounds.

## Author Contributions

M.N., N.P. and G.A. designed the experiments. M.N. performed the experiments and collected data. S.H. and G.A. discussed the pathology score results. N.P. supervised, directed and managed the study. All the authors approved the final version to be published.

## Funding

The authors have nothing to report.

## Ethics Statement

This study was approved by the National Committee on Ethics in Biomedical Research (ethics.research.ac.ir) of the Islamic Azad University, Science and Research Branch (approval ID IR.IAU.SRB.REC.1401.339). Moreover, in accordance with the Helsinki Declaration, this research was conducted with due care to protect the welfare of the animals involved.

## Conflicts of Interest

The authors declare no conflicts of interest.

## Data Availability

The data presented in the study are included in the article, along with additional material.
